# Educate Kermit Roosevelt Through Sport Hunting and Train Him for Government Missions. Roosevelt Scientific Mission to English Equatorial Africa in 1909

**DOI:** 10.3389/fspor.2021.638528

**Published:** 2021-10-18

**Authors:** Romain Chasles

**Affiliations:** The Institute of Sports Sciences of the University of Lausanne (ISSUL), Faculty of Social and Political Sciences, University of Lausanne, Lausanne, Switzerland

**Keywords:** sport hunting, military sport, legacy, english empire, Theodore Roosevelt, postcolonial studies, socio-history

## Abstract

*The practice of sport hunting in colonized areas presents a set of knowledge and techniques indispensable to self-control and the domination of territories elsewhere by colonial empires, by their leaders and, more generally, by the political elites of the Northern states*. During his scientific mission to English Equatorial Africa in 1909, Theodore Roosevelt responded to a double commission from the Smithsonian Institute and the American Museum in Washington. In this African mission, he brought and trained his youngest son Kermit, aged 20, in an initiatory journey. This article proposes to study this ritual of passage and the practice of sport hunting in the English colonial space as a revelation of the socio-racial hierarchies at work in the territories dominated by the English Empire.

## Introduction

Hunting and politics have a deep relationship. The hunting practice is framed, legislated and controlled by politics. At the same time, it is also an activity prized by the socio-political elites when it comes to hunting in faraway places, a privilege that the aristocrats and high bourgeoisie of the North retain in these spatial and temporal frameworks.

For the contemporary period, the European opening to the conquest and colonization of African and non-Western areas generates a displacement of cultural practices in these spaces and certain forms of hybridization of physical practice in contact with the colonized other. Under these conditions, the practice of sport hunting in the colonized areas presents a set of knowledge and techniques essential to the self-control and the domination of the territories by the colonial empires, by their rulers and, more generally, by the political elites of the Northern States.^1^ The practice of sport hunting gathers scattered age categories although its government is most often made up of central personalities, where the presuppositions related to social recognition, field and practice experience, and maturity are mixed^2^.

## Materials and Methods

Historiography has long dealt with the establishment of monographs on the life and work of Theodore Roosevelt Jr (Morris, 2002, 2010; Pringle, 2003). The 26th President of the United States also appears as a political strategist, an avant-garde environmentalist and an informed explorer while being a recurring and insatiable sportsman (Zachary, 2012; Marquis, 2014). In these monographs, his relationship with his son, Kermit Roosevelt, during the 1909 mission, however, is neglected. Nevertheless, it reveals essential points of his life: a deep training in sport hunting, the torments of the missions and a family transmission of knowledge and government techniques from father to son.

During his scientific mission in Equatorial Africa and under English protectorate in Kenya, Uganda, Sudan and Egypt in 1909, Theodore Roosevelt responded to a double commission from the Smithsonian Institute and the American Museum in Washington. He brought and trained his youngest son Kermit, aged 20, in this African mission. Theodore Roosevelt and Kermit Roosevelt share a common pleasure for physical and sports activities in the great outdoors and for explorations (Enders, 1998)^3^. As he prepares to enter Harvard, Kermit follows his father's journey on his “tour” of European governments and his African mission. However, what does this rite of passage reveal in the training and education of young Kermit by his 50-year-old patriarch Theodore Roosevelt?

The practice of sport hunting in the English colonial space is a revelation of the socio-racial hierarchies at work in the territories dominated by the English empire. How does the young Kermit Roosevelt fit into this process of mission government and hunting practice?

Sport hunting also shows a different relationship to age than other physical and sporting practices. This difference of age and generation is even more marked as the experience and the age of the person in charge of the mission are important. He imposes an initiation to the young practitioner in front of the risks inherent to the practice. In particular, since he trains his sons in extra-national and imperial spaces, which are supposed to be less mastered by Theodore Roosevelt. Also, this practice cannot take place without a tutelary figure and a complete organization—borrowed from the military hierarchy—to guide the young sport hunter in the learning of hunting techniques and the government of the missionary organization. Does Kermit Roosevelt's closeness to his father engage him in the role of a missionary second, despite his young age and the presence of other experienced sportsmen? How does Kermit Roosevelt position himself in the government and hierarchy of the mission?

The historiography of colonial hunting, from the beginning of the 19th century to the independence of the former colonial territories, has insisted on the role of marking the cultural and sporting practice of hunting in the domination of the colonized territories, in the appropriation of indigenous practices by the colonial elites and in the globalization of sport from the North to the South (Singaravelou, 2011). It has also provided convincing results on the metropolitan culture that stems from these cultural practices around the heroic figures of the first sportsmen and explorers in the colony, a kind of constant mimicry to the reiteration of the colonization of spaces elsewhere (Mackenzie, 1984). The globalization of sport is a phenomenon that takes on its full meaning in the colonial space (Combeau-Marie, 2006). It even accelerates the dynamics of colonization and domination against colonized groups (Bouvier, 2010; Hussain, 2010, 2012).

The historiography of colonial hunting, as a culture proper and inseparable from imperial logics, also reveals logics of reiteration of racial and gender hierarchies in practice. The masculine territory of hunting practices - in this case of colonial origin—creates de facto spaces of exchange between men from which women are excluded (Steinhard, 2006). The masculinist culture of the hunting elites is reinforced by these international sports trips. Within these male hunting cohorts, racial hierarchies are deconstructed and confirm the centrality of this practice in imperial culture (Mackenzie, 1984). In contrast to women, the colonized hold a central place, but one that has always been orchestrated by cosmopolitan elites. The relationship between the military and the sport of hunting in the colony reinforces the logic of domination toward the colonized. It assigns them to subordinate positions in the practice of hunting, excluding them from spaces and practices (Roulet, 2004). Therefore, this sport hunting culture is accompanied by an ethic specific to its practitioners (Michaud, 2010; Malarney, 2020), which contributes to the construction and permanence of this elite “entre-soi” (Bourdieu, 1989).

As a sporting practice of a young hunter in a central scientific mission for the United States, are the honor and experience framing the hunting oriented toward a less intensive and less dangerous quest than that of his father? In other words, does Kermit Roosevelt's young age engender a less sporting inflection of his hunting practice? Is this process sustainable during the mission?

This work therefore proposes to study the travel narrative and the collections collected by Theodore Roosevelt and Kermit Roosevelt for the Smithsonian Institute and the American Museum in Washington to explore these questions^4^.

This article aims to contribute to the research of cultural history and socio-history in the African colonial space. Few studies have been conducted on the colonial field for socio-history even though its research prism directs it toward questions of domination and interdependent networks (Elias, 1981). The digital archives of the Smithsonian Institution, the Theodore Roosevelt Center, and his book allow us to follow the thread of the Roosevelts' mission in the colony according to a classic reading of the historical archive (Venayre, 2006; Delmas, 2017). This book also holds a central place in our study in that it relates the day-to-day peregrinations of the Roosevelts in the English African colony as a sort of logbook from which it results. Furthermore, and despite being a literary object, the analyses conducted in this article take the bet that the writings delivered in this travel document are at least the fruit of effective hybrid representations of Theodore Roosevelt (Roux, 2012). More than that, they are dynamics at work during the Roosevelts' mission. They are embodied in the formation of the young Kermit Roosevelt on mission and after his journey.

## Results and Discussion

### Train the Young Kermit Roosevelt in the History of Hunting, the Equipment and the Organization of the Hunting Mission in English East Africa

#### Build a Library and Discover the Hunting Books as an Introduction to Sporting Activities

The hunting practice of the young Kermit Roosevelt in the colony is based on literary knowledge whose acquisition prepares and exceeds the very framework of the sporting experience. Their role is to be understood as an *actant* in the hunting mission that occupies the Roosevelts in that “*the books were stained with blood, sweat, gun-oil, dust, and ashes”*^5^ (Latour, 1990). Thus, the books have a life outside and within the Roosevelt's journey. They act on the mission of the Roosevelts and experience their practices. During his European and African journey, Theodore Roosevelt carried his pigskin library, and several books added for the occasion, which he shared with his son as milestones in his intellectual and athletic training (Chasles, 2015). This literary knowledge guided Kermit Roosevelt's practice in the English African colonies during 1909:

“There was one other bit of impedimenta, less usual for African travel, but perhaps almost as essential for real enjoyment even on a hunting trip, if it is to be of any length. This was the “pigskin library,” so called because most of the books were bound in pigskin. They were carried in a light aluminum and oilcloth case, which, with its contents, weighed a little <60 pounds, making a load for one porter.”^6^

This literary travel experience, in which the burden of transportation is given to a colonized bearer, is innovative in the register of works relating to colonial hunts. In fact, it goes beyond the references that colonial hunters develop in their retrospective essays. Indeed, they linger, most often, on the essays that guided them empirically in their travels^7^.

However, even if the only tangible and verifiable trace is the transport of this pigskin library in Africa, we can assume that these books are anchored in long-term knowledge and that their transport should form Kermit Roosevelt in the long term. They are also a reminder of the importance of their understanding for the present mission. This training takes place in a quartet of literary choices made by his father: “the Bible, Shakespeare, Homer, and Dante” in which international classical literature is mixed with Christian biblical texts^8^.

More than any other sporting and physical practice, hunting in a colony is part of an obligation to master a theoretical and naturalist knowledge because it obliges the hunter to determine and think: the organization of his trip, the space in which the hunts take place, the animals hunted (Ford et al., 2016). The acquisition of knowledge specific to hunting also raises the question of the use of the results of the hunts, which can be of several kinds. These may be hunting trophies, hunting records or colonial circulations which are still, at the beginning of the 20th century, a source of central interest for Geographical societies. Obviously, these results are a central source for states with regard to geopolitical and imperial issues. As for them, hunting trophies and records respond in our case study to orders from public institutions and to more personal needs of the Roosevelt family. Thus, Kermit Roosevelt's knowledge, acquired and in the process of being acquired, guides his colonial hunts through a game of back and forth between theoretical and practical learning. As the title of Theodore Roosevelt's book confers, the theoretical knowledge at stake in this mission largely concerns naturalist science and hunting in a constant relationship. Finally, literature and its learning are at the center of Theodore Roosevelt's training as reported in the various articles and questions on the pigskin library^9^.

The Roosevelt readings create a space of instruction, representations and a field of possibilities for the practice of hunting in the colonial space studied^10^. As such, they guide the practice and determine its framework. In this regard, Theodore Roosevelt stipulates that:

“It represents in part Kermit's taste, in part mine; and, I need hardly say, it also represents in no way all the books we most care for, but merely those which, for one reason or another, we thought we should like to take on this particular trip.”^11^

The pigskin library and the Roosevelt's additional works are the result of a deliberate choice undertaken by Theodore and Kermit Roosevelt on the occasion of their mission, without being exhaustive. Theodore Roosevelt admits, however, that it could evolve if the mission were to be reiterated or changed. Sharing the travel library with his father allows us to think about the different exchanges between the two Roosevelt's and their entourage^12^. The Roosevelt's library was enriched during the trip with some reading tips from guests and meetings held in Africa during the mission. Former President and agent of the first world power, the Roosevelt's arrival in Europe and during this imperial African mission represents, in 1909, a real knot of sociability during which more personal and cultural issues are addressed. The weight of literary issues is central here and also testifies to the inclination of American elite culture for European literature. It also strongly affirms the connections between the world's cosmopolitan elites (Cotesta, 2011; Frank, 2014).

The books kept in the Roosevelt's travel library provide general references to the hunting experience and to the elite literature, known as classical. For example, Goethe's initiation novel Faust is addressed to Kermit Roosevelt^13^. Its presence reveals the prerogatives of the young Roosevelt's training to be perfected. It provides information on the learning to be developed during his journey. Finally, Faust could help Kermit Roosevelt's self-construction, his passage to adulthood. Faust actually confirms Theodore Roosevelt's ambition toward his son regarding the African mission, showing the rite of passage to be carried out during their epic. His reading also participates in the permanence of the elitist self-segregation in which the young Roosevelt must maintain himself. The collection of international poetry, present in the corpus, confirms this desire for continuity in the formation of Kermit Roosevelt. They also serve as a support for romantic reveries and travel fantasies. Theodore Roosevelt states as follows:

“Often my reading would be done while resting under a tree at noon, perhaps beside the carcass of a beast I had killed, or else while waiting for camp to be pitched; and in either case it might be impossible to get water for washing.”^14^

The body of books also shows a considerable literary appetite for European history, be it prehistoric, ancient or more modern. It also includes books of the Enlightenment and biographies of royalty as well as historical essays on the history of European states^15^. As confirmed by the titles of the books, they also denote the ability of the Roosevelt family to read books in French, further proof of the insertion of this family in international and especially imperial matters.

The choice of certain texts reveals a precise hunting experience and a finer choice, relating to the particularity of the mission and the needs generated by it^16^. The references directly related to hunting take place in a corpus of scientific, travel and political books ranging from Darwin's *Origin of Species* to Gobineau's *Inégalité des Races Humaines*. We could even add that these works are part of the imperial and cosmopolitan hunting culture. We propose to focus on a central example. *Tartarin de Tarascon* seems indeed to reflect this symbiosis between the training of the young Kermit and the sharing of an international hunting culture^17^. This pedagogical corollary tends to confirm the interdependent relationship between youth training and hunting in this elite cosmopolitan world.

““Tartarin de Tarascon ” (not until after I had shot my lions !).”^18^

With these few words, the temporal veracity of which cannot be proven - the travel narrative reconstructs the times of the mission—Theodore Roosevelt affirms the centrality of the novel within his mission while delivering some humorous notes to his readers on the hunting universe and its excesses. That said, the presence of Alphone Daudet's book sets out the cultural framework specific to hunting, which itself refers to a white African hunter of the 19th century: Jules Gérard (Venayre, 2002). This quotation also allows the author to re-establish the supremacy of one type of African hunting over the others, namely the hunting of lions. By taking advantage of the scientific mission that institutionally occupies the Roosevelts, lion hunting presents an obligatory step to enter the cosmopolitan circle of elite hunters, and this is what the two Roosevelts are dealing with on their travels^19^. It is no coincidence that this hunt is presented first. Indeed, it takes a special place in the Roosevelt's hunting story. This literary operation makes explicit the use of the scientific mission with more individualized purposes for the Roosevelts.

This set of volumes is one of the theoretical and cultural foundations of the practice of international sport hunting, a sort of cement and shared references as to the prestige and seniority of the hunters. Moreover, Kermit Roosevelt's literary and theoretical training in colonial hunting is also based, in a symbiotic way, on a theoretical and practical knowledge of the weapons and equipment inherent to the practice of hunting. Kermit Roosevelt's father considered literature and weapons to be of equal importance. As such, if there were no choice with regard to these two needs, it would become impedimenta for the mission^20^.

#### Build Up an Arsenal of Rifles and Equipment to Prepare for the Hunting Ground

Kermit Roosevelt's arsenal of firearms and equipment reveals both his learning of the practice of sport hunting and his dense integration into the cosmopolitan hunting environment. Indeed, the act of hunting seems intimately linked to the transport of hunting equipment by the Roosevelt family. More than the transport of this equipment, which constitutes an ordeal in itself and which can lead to a scientific study in its own way, we will try to understand what the material traces are conveyed by Kermit Roosevelt and his father. These have a noticeable influence on the establishment of cultural models in the new spaces being practiced. Thus, it is easier to understand this history by its visible character that responds directly to the different senses in action of the hunters. We will use Theodore Roosevelt's pointed explanations of rifles, shotguns and hunting equipment to unravel the concepts which emerge from the use of these key hunting objects.

The artillery on the Roosevelt mission camps is representative of the central role of this equipment in hunting trips. Like the Roosevelt library, their charge is entrusted to a designated carrier. The arsenal of weapons refers first of all to knightly representations. In the same way, it corresponds to the question of the choice of weapons which individualizes the sportsmen as much as it incorporates them into the colonial hunting culture. The writing of the book allows us to imagine the weight of the *pater familias* in the choice made by Kermit Roosevelt. It also reveals differences linked to the hunting experience and the weight of the individualization of the young Kermit in his practice. The description of these weapons then makes it possible to differentiate the levels of insertion of the two Roosevelt's in international hunting. Their comparison shows the level of training and integration of Kermit Roosevelt in the hunting environment.

From a literary point of view, the arsenal of rifles is most often brought to light in the beginnings of books on hunting trips, which reiterates its centrality. This also accentuates the narrative on the romantic and dangerous quest that hunters lead (Hahn and Bouquet, 2014)^21^. The travel narrative, whose publications were central during the 19th century, thus responds to codes of writing aimed at a defined public. It also tends to present to the most knowledgeable the level of organization and choices developed by the Roosevelt family, which is part of a double game between the collective tradition of the self and individual innovation of differentiation. Theodore Roosevelt respects this historical framework for the presentation of travel narratives, which reiterated his family's insertion into the group of international hunters.

Like the library and the literary issues mentioned above, the Roosevelt arsenal is first and foremost a place of sharing where the transmission of knowledge and empires from Theodore Roosevelt to Kermit Roosevelt takes place. But what is this sum of arms? What does it tell us?

“My rifles were an army Springfield, 30-caliber, stocked and sighted to suit myself; a Winchester-405; and a double-barreled 500–450 Holland, a beautiful weapon presented tome by some English friends. {…} In addition, I had a Fox No. 12 shotgun; no better gun was ever made”^22^.

This sum of arms shows their role as actants in the Roosevelt's mission in colonial Africa. It is even more so because these guns come from the military world. The choice of Roosevelt rifles thus confirms the proximity of the practice of sport hunting with the military, especially since this mission took place in imperial Africa. Does Theodore Roosevelt see in this trip the possibility of perfecting his son's military education or even of giving him an essential part in his training? In any case, this mission represents an opening up of the field of possibilities in terms of complementary instruction in the choice of arms, their handling and their use by the young Kermit Roosevelt. The general arsenal of the Roosevelt family reveals the predominance of the *Winchester-405*. Also, the Roosevelt used American rifles—the *Winchester 405* from New Haven (CT) and the *Springfield 30* caliber from Madison (NC)—more than usual to ensure the dominance that the American arms industry could obtain from the political and hunting elites in the African space. On this point, only the Holland 500–450 rifle seems to stand comparison in terms of the capacities it gives to hunters in the *big game*.

“The Winchester and the Springfield were the weapons one of which I always carried in my own hand, and for any ordinary game I much preferred them to any other rifles. The Winchester did admirably with lions, giraffes, elands, and smaller game, and, as will be seen, with hippos.”^23^

During the Roosevelt's mission, these firearms were therefore used and thought of as generalist rifles that proved their adaptability, their generalist aspect and their potential predominance in the practice of sport hunting. As for them, the *Fox n*°*12* rifles, from the Baltimore military industry, are used for birds and waterfowl. The generalization of American armed equipment during the last conflicts of the 19th century contributed to the diffusion of these weapons on the international market. Thus, this spread has also allowed a dissemination of these guns in international elite circles and, in this case, in the practice of hunting in Africa.

The general arsenal of the Roosevelt family also suggests an individualization of the practice of the firearms by Kermit Roosevelt.

“Kermit's battery was of the same type, except that instead of a Springfield he had another Winchester, shooting the army ammunition, and his double-barrel was a Rigby. In addition, I had a Fox No. 12 shotgun; no better gun was ever made.”^24^

Therefore, he borrows the choice of guns from his father. He also added the *Rigby double barrel*, an elite weapon from the Irish military industry that became a distributor of Mauser rifles for the British Empire. This manufacturer targets both military and civilian circles so that we can understand its use by Kermit Roosevelt.

“Kermit on this occasion was using the double-barrelled rifle which had been most kindly lent him for the trip by Mr. John Jay White, of New York.” ^25^

The latter uses here the double Rigby gun against a rhinoceros of which he loses the trace before finding it again about 10 days later. Apart from this weapon, Kermit Roosevelt is equipped with the Winchester, a simpler and more generalist rifle unlike the Holland-Holland and the Springfield rifle. The whole arsenal of weapons entrusted to Kermit shows his difference from the hunting community he is trying to fit into. It shows the experience necessary to fully integrate the hunting culture.

Finally, the set of hunting objects present in the hunting camp and used by the Roosevelt reveals the predominance of the military model in their peregrinations in Africa^26^. In addition to this, there is also equipment entrusted by big-game hunters to the Roosevelt. This omnipresence reinforces our hypothesis as to the simultaneous transmission to the young Kermit Roosevelt of a hunting and military culture.

To the group of actants present during the Roosevelt's African mission, a sum of human actors is added, forming a network around Kermit Roosevelt.

#### Coach Kermit and Build a Group Around Themselves to Hunt in the English Colonial Empire

During his colonial journey, Kermit Roosevelt received training in government and hunting (Dimier, 2004). To do so, he is surrounded by a group of colonials and hunters who transmit to him the knowledge indispensable to his physical and intellectual education. Three groups of men seem to stand out among the cohort of Westerners. The first one comes from the high social and political cosmopolitan spheres, way to the learning of international politics and diplomacy. These groups help the Roosevelt in their circulations, mostly in the English colony. He participated in the military and imperial training of Kermit Roosevelt. The second brings together scientists who are supposed to accompany Kermit Roosevelt in his naturalist apprenticeship and in the different approaches to African ecosystems. The third brings together experienced hunters who are close to the hunting practice, advising and supervising Kermit Roosevelt's hunting practice.

In a way, the aim is to set up the Prince's court in order to participate in his training on the African field. Halfway between the aristocratic European Great Towers and the Renaissance education advocated by Erasmus, Kermit is framed in his African journey by a sum of networked actors (Bertrand, 1999; Boutier, 2004). Like a Prince of Italy of the cities of the modern era, it is important to give the necessary and remaining instruction to his cosmopolitan elite education, especially since his age corresponds to this rite of passage into adult life. This pedagogical process may show, without this being expressly stated by Theodore Roosevelt, a choice made by his father in the training of the young Kermit in imperial and diplomatic matters. Perhaps, more or less consciously, he had made Kermit Roosevelt his second in order to train him in politics? It is moreover central to note that Kermit Roosevelt had joined the prestigious Harvard University a few months earlier^27^. In exchange for participating in his father's mission, Kermit Roosevelt was promised the opportunity to take a two-year crash course at Harvard to complete his degree. The actors present around Theodore Roosevelt's son during the scientific mission are to be understood as a network (Bloor, 1991). Kermit Roosevelt could be considered as the second atom of the African mission where the different actants are of interest. They create their own singular configuration (Elias, 1993).

The first category with which Kermit Roosevelt has ties that build his education could be conceptualized as the international socio-political high spheres. Although the latter is restricted by the journey itself, this international horizon - note the absence of connections with French colonial elites and administrators—the position of the English empire in the world sets some of the imperatives for the Roosevelts to travel there. Moreover, the imperial development of English territories combined with the structuring of the practice of hunting sports in these spaces constitute one of the two fundamental reasons for the establishment of this mission in English colonial Africa. The high spheres, at the center of the Roosevelt mission, are catalysts for the supervision of Kermit Roosevelt during his mission. Sir Edward Gray and Lord Crewe, respectively English Foreign Secretary and Leader of the House of Lords, facilitated the smooth progress of the Roosevelt's trip and ensured that he learned about imperial affairs in the same way as the Belgian Minister for the Colonies, Mr. Renkin^28^. The same goes for philanthropist and industrialist Andrew Carnegie, former Secretary of State for Commerce Oscar Straus, and entrepreneur Leigh S. J. Hunt, who are closely involved, as donors to the mission, in the organization and success of the Roosevelt expedition to Africa. We do not focus here on the interactions generated by the Roosevelt's European tour and in the preparation of the mission where Kermit Roosevelt was present with his father. In the field, Sir Alfred Pease played a preponderant role in this transmission of imperial and political powers. Formerly elected to the House of Commons by the Liberal Party, he influenced Kermit Roosevelt's movements in the colony. Author of several scientific articles, Alfred Pease is best known for the second part of his career when he became one of the main pioneers of English East Africa^29^ (Storey, 1991).

“Sir Alfred and Kermit were tearing along in front and to the right, with Miss Pease close behind, while Tranquility carried me as fast as he could on the left, with Medlicott near me.”^30^

Second Baronet to Hutton Lowcross and Pinchinthorpe, he was the son of Sir Joseph Whitall Pease, a Quaker entrepreneur and was educated at Trinity College in Cambridge. He is also a past member and President of the Cleveland Bay Horse Society. The second part of his life was occupied with the imperial question in Africa, residing in South Africa where he was a trustee and then moving from North of the Sahara to English East Africa where he chose to settle a short distance from Nairobi to set up an ostrich ranch and practice hunting. During the Roosevelt's mission in Africa, Sir Alfred Pease will be an initiator for Kermit Roosevelt to the imperial object. During the course of the mission, they usually form an immovable couple as to better transmit to the young Kermit the experiences necessary for his training, especially since Sir Alfred Pease represents the cumulative figure of experience, the established settler and the seasoned hunter^31^. Moreover, this man resembles Theodore Roosevelt while allowing him to disengage himself from him.

The second category with which Kermit Roosevelt maintains strong relationships during its mission is the scientists who belong to the Smithsonian Institute's mission. Among them, Dr. Edgard A. Mearns, Andrew Carnegie, Oscar Straus, and Leigh Hunt are the most prominent.

“That night Kermit and Dr. Mearns went out with lanterns and shot guns, and each killed one of the springhaases, the jumping hares, which abounded in the neighborhood. These big, burrowing animals, which progress by jumping like kangaroos, are strictly nocturnal, and their eyes shine in the glare of the lanterns.”^32^

An American military surgeon and ornithologist, Dr. Mearns leads the scientific part of the Roosevelt mission in English colonial Africa^33^. Initially engaged for 25 years in the U.S. Army until 1909, he chose to continue the work of ornithologist and herbalist that he had begun 10 years earlier on Mexican territory collecting plants and species for the United States National Museum. Dr. Mearns is therefore a truly experienced guide for Kermit Roosevelt although the African territory is new to him^34^. He was the group's best trigger man, and together with Alden Loring, he formed a formidable team of collectors helped by the young Kermit^35^. Meanwhile, Alden Loring is a naturalist scientist, specializing in the study of mammals. He has already participated in collecting expeditions in Europe and Scandinavia. He was able to collect a small thousand mammals before his departure for Africa (Appetiti, 2009). He is well-versed in scientific practice and in international expeditions, and his proximity represents an aid and a man of knowledge for Kermit Roosevelt. In addition, these two men are assisted by the American zoologist and naturalist Edmund Heller. The latter is a seasoned adventurer, having already participated in expeditions to the Galapagos Islands, Mexico, Guatemala and Alaska^36^. This triptych of three American scientists is part of the Roosevelt's African mission, as it is a response to a commission from two institutions. This group participates very largely in a heavy trend of exhaustive collections in the African field in which Kermit Roosevelt is largely involved. It is indeed a question of participating in the greatness of the nation while collecting all the forms of knowledge relating to the naturalist science lavished by these men of the field.

The third category that Kermit interacts with during his African journey is colonials and seasoned hunters. This group of colonial hunters exhibits the dense links between understanding the hunting terrain and experienced mastery of his hunting practice. Within this group of colonial hunters, Frederick Courtney Selous and E. N. Buxton whom the Roosevelt's found on their boat leaving Naples for East Africa.

“Selous; and, so far as I now recall, no hunter of anything like his experience has ever also possessed his gift of penetrating observation joined to his power of vivid and accurate narration. He has killed scores of lion and rhinoceros and hundreds of elephant and buffalo; and these four animals are the most dangerous of the world's big game, when hunted as they are hunted in Africa. To hear him tell of what he has seen and done is no less interesting to a naturalist than to a hunter. There were on the ship many men who loved wild nature, and who were keen hunters of big game.”^37^

Although absent from the Roosevelt's African field mission, their empirical experience and their books sharpen the Roosevelt's peregrinations and hunts. We understand here that the weight of narration and orality is important within the hunting culture. The presence on the Roosevelt cruise ship of these two white hunters is far from being fortuitous (Herne, 2001). On the contrary, they participate in the first training of Kermit Roosevelt in the throes of the colonies and the practice of African sport hunting. Regarding Mr. Richard John Cuninghame, Mr. Harold Hill and Mr. Leslie Jefferies Tarlton, they are all three long time colonial, military and naturalist (Brown, 2004). Their work with Kermit Roosevelt consists of advising and anticipating the Roosevelt procession in Africa by opening the expedition's tracks and responding to all the imponderables of the mission. All three are known to be big-game hunters, their experienced knowledge of the ground via expeditions in English East Africa and/or their service during the Boer War allow the smooth running of the mission^38^. This supervision by professional hunters benefits the young Kermit and facilitates the collective quest related to the Roosevelt mission. Theodore Roosevelt even considers that Richard John Cuninghame is the manager of the mission^39^.

### Sharing a Sport Between Father and Son in Colonial Africa: A Practice of Progressive Emancipation?

#### The Scientific Quest of the Mission: Hunting to Collect

Kermit Roosevelt's practices in the colony corresponded to the first scientific and colonial order his father received. This order defined the framework of the Roosevelt's activity in the colony and was to serve the entire American nation.

The Roosevelt's mission was first to respond to a scientific commission from two American public institutions.

“On March 23, 1909, I sailed thither from New York, in charge of a scientific expedition sent out by the Smithsonian Institute, to collect birds, mammals, reptiles, and plants, but especially specimens of big game, for the National Museum at Washington.”^40^

This mission demonstrates a desire to assert the power of the American nation on extranational soils. This American purpose responds to a recent inversion of the Monroe Doctrine via the Roosevelt Corollary, of 1904, pronounced by Theodore Roosevelt (Compagnon, 2009). This inflection of foreign policy tends, among other things, to be corroborated by this scientific mission to Africa. Indeed, it is not only a sporting practice innocently chosen and carried out on these territories dominated by the English empire. It is about participating, under the leadership of the former first representative of the American people, in the procession of the imperial nations, and of the first colonial empire in this case. The American extroversion permitted by the Roosevelt mission in English colonial Africa opens, in fact, to the inflection of the USA on the geopolitical, diplomatic, scientific level and on a desire for elsewhere and connections beyond the pre-square of Latin America. We do not focus on studies of the American people's reception and perception of this mission. However, the media do participate in this change in representations of the geopolitical role of the US, at least for the American elites. The mission opens up a new international horizon for the U.S. In contrast, we do not forget either that this mission of the Roosevelt's gave rise to cartoons and caricatures about the Roosevelt's, proof of the weight of the reception of this mission on American soil (Harbaugh, 1975)^41^.

This triple play between international diplomacy, scientific research and African hunting reinforces the three themes simultaneously. Indeed, we understand that as scientific research develops, the field of possibilities for diplomatic exchanges opens up. In addition, the Roosevelt's are supported during their mission by the Smithsonians who reiterate the link between scientific command and hunting practice in the field.

“On this first five weeks' trip we got over 70 skins, including 22 species, ranging in size from a dikdik to a rhino, and all of these Heller prepared and sent to the Smithsonian.”^42^

The Roosevelt and their cohort of professional African hunters were entrusted with the most famous scientific hunts. As the hunts of the two Roosevelt's proceed, the naturalists involved in the mission participate in the categorization and preparation of the animals killed by Kermit and Theodore Roosevelt. Given the magnitude of the animals collected, shipments to the U.S. are spread out over the entire mission. We hypothesize here that these shipments allow the American scientists on site to begin the first analyses of the animals and samples of all types received. They also allow their faster museographical presentation within the African collections of the National Museum of Washington^43^. In fact, the Museums of major international capitals are competing for their collections. The Smithsonian Institute's funding of the Roosevelt's African mission is therefore not philanthropic (Mackenzie, 2010). It is a museum that affirms the place of the United States in this international competition.

“Few laymen have any idea of the expense and pains which must be undergone in order to provide groups of mounted big animals from far-off lands, such as we see in museums like the National Museum in Washington and the American Museum of Natural History in New York.”^44^

The United States joined the imperial, museographical and scientifical competition despite its lack of colonial territories in Africa. The Roosevelt's commitment in Africa even supports, in a consequent manner, the present scientific mission, given the rank and fame of Theodore Roosevelt and his family. It is then a question of bringing the United States up to standard by the effect of the Roosevelt's hunting practices. Apart from the animals hunted for the venison that supplies the Roosevelt porters and aides-de-camps, the Roosevelts are busy participating exclusively in the construction of the African wildlife collection^45^. Faced with an innumerable fauna that corresponds to as many fantasies of the white African hunter as to an empirical reality, the Roosevelt's reduce their shooting in order to realize the collection that was given to them. The objective of the Roosevelt hunts is indeed the completion of this collection with a scientific and imperial aim. It shows a hierarchy of values and potentialities generated by the mission. This goal places even the Roosevelt's in the midst of sports hunters refusing international sporting competition. Theodore Roosevelt even compares them to autograph collectors and philatelists^46^. The Roosevelt hunts reveal the supremacy of scientific purpose over all other forms of hunting. This represents an inalienable character of the African mission.

The activities of Dr. Mearns and Alden Loring confirm the scientific orientation of the Roosevelt quest. The Roosevelt's are at the heart of the mission. Their practices of sampling and collecting small mammals, birds and African flora punctuate the times of the mission. Thus, the naturalist sampling and collection exercises force the rest of the caravan to wait for them so that they are complete.

“Often, while Heller would be off for a few days with Kermit and myself, Mearns and Loring would be camped elsewhere, in a region better suited for the things they were after. While at Juja Farm they went down the Nairobi in a boat to shoot waterbirds and saw many more crocodiles and hippo than I did. Loring is a remarkably successful trapper of small mammals. I do not believe there is a better collector anywhere. Dr. Mearns, in addition to birds and plants, never let pass the opportunity to collect anything else, from reptiles and fishes to land shells. Moreover, he was the best shot in our party.”^47^

Dr. Mearns and Alden Loring are key to understanding how the mission will unfold. In fact, each move and parking on spaces requires them to be exhaustive in their collections. The former is more active in killing and collecting birds, reptiles, fish and mollusks, while the latter sets traps for small mammals. Their roles in the mission correspond to their skills already deployed in the various naturalist missions in which they have been able to participate.

As said, the Roosevelt's and their escort show a sense of accountability to the nation and to American scientific institutions. Therefore, the commitment of the mission follows this shared goal. In addition, the African mission has received additional support in its funding from U.S. philanthropists willing to participate in this national effort^48^. Within the scientific framework setting the mission's travel and collection rules, the Roosevelt's hunting practices can be understood as the foundation of an extroverted, extraterritorial nationalism. They are a support for its expression outside American territory and in the eyes of African imperial issues.

#### Hunting With the Family to Learn How to Play Sports on the African Field

The Roosevelt hunts in camp are a privileged moment to share a sport practice with the family. They are a moment of transmission through the practice from father to son. These hunts are also spaces of constant demonstration for Kermit Roosevelt in order to signify to his father of his passage in adult life.

The Roosevelt's hunting practices during the one-year African mission are therefore multiple. They denote an excess in the hunting demonstrations of the two leaders, among others, of the mission. On the one hand, it is a question of killing big game, the noblest for the big-game hunter, the one that also has the most important scientific and museographic value. Unusually, the Roosevelt's African hunts are mostly conducted on horses, which shows the hybridization of African hunting practices. Small horses that are compared to zebras for their size and whose charge is given to aides-de-camps^49^.

“Our hunt after wildebeest this afternoon was successful; but, though by veldt law each animal was mine because I hit it first, yet in reality the credit was communistic, so to speak, and my share was properly less than that of others. I first tried to get up to a solitary old bull, and after a good deal of maneuvering, and by taking advantage of a second rain squall, I got a standing shot at him at 400 yards, and hit him, but too far back. Although keeping a good distance away, he tacked and veered so, as he ran, that by much running myself I got various other shots at him, at very long range, but missed them all, and he finally galloped over a distant ridge, his long tail switching, seemingly not much the worse. We followed on horseback, for I hate to let any wounded thing escape to suffer. But meanwhile he had run into view of Kermit; and Kermit who is of an age and build which better fit him for successful breakneck galloping over unknown country dotted with holes and bits of rotten ground—took up the chase with enthusiasm”^50^.

The study of the Roosevelt family's hunting practices reveals their ongoing support for each other. It demonstrates patriarchal relationships that existed in the mission. Here, Kermit Roosevelt is given the responsibility of finishing his father's hunting job by chasing a wounded Wildebeest. This support is even stronger since the fruit of the hunt is always left to the one who first victoriously shoots the prey. This spirit of hunting camaraderie flows throughout the entire African mission. It is supported by the hierarchy of the mission. Like an army on the move, the established organization of the mission internalizes the precise role of each actor in the mission. In addition, the beginning of the mission shows a strong filiation of Kermit Roosevelt to his father. When the latter chooses to shoot, he is followed by his son for a few seconds. The experience in hunting practices of Theodore Roosevelt tends to show, through example, the practices to be carried out by his son.

Conducted on African lands in English East Africa, the hunting practices increase the variety and learning of hunting techniques of the young Kermit. In contact with his father and experienced hunters, Kermit Roosevelt learns by practice and demonstration from the experienced majority within his missionary cohort. These empirical practices make the link with all the theoretical knowledge-powers previously analyzed (Foucault, 1975). This can be understood as a continuous relationship between hunting theory and practice. On horseback or on the look-out, sometimes accompanied by dogs, the Roosevelt's hunts target all the big game of the East African English ecosystems. When these hunts are on the look-out, Kermit Roosevelt and Theodore Roosevelt are positioned by the retreaders at the ends of the shooting range so that, depending on the movements of the animals, their shooting returns to one or the other^51^. These techniques of placement and reeling are profitable to the Roosevelts, as if to reiterate their central role in the mission with regard to the most famous animals of the African fauna. The diversity of the Roosevelt hunts also shows the avalanche of fire when it comes to tracking mammals living in groups such as buffalo, various antelopes or wildebeests. These continuous shootings make it possible to reconsider both the place of sound in these African hunts and the place of individuality in hunting practice (Hemery, 2006). However, we note that this multiplication of shooting is not aimed at the top of the African hunting pyramid for reasons specific to these species. Considered big game, elephants, rhinoceroses and buffaloes are thus hunted in groups by the Roosevelts^52^. On the other hand, the pomp conferred on lions, leopards and cheetahs directs hunters toward more individualized hunting practices. They show more personal objectives specific to the cosmopolitan hunting community. Among the most famous big game species, the lion is obviously the most significant one. It refers to the myths and fantasies of the first African explorations and colonization but also to the tangible dangers on these territories (Thompsell, 2015)^53^. The hunting practices of the Roosevelts participated in the visible inclusion of Americans in African imperial history. They give way to precise considerations and judgments as to the hunting order established by the hunting community, without, however, taking into account the facilitated preparation of these hunts^54^. In any case, the hunting of lions and various carnivores occupies a large part of the Roosevelt hunts in Eastern colonial Africa. It is a crucial stage, an obligatory rite of passage, for Theodore and Kermit Roosevelt, opening the first big game hunts of the story. The orientation of the expedition records from the first weeks, a direction toward this wildlife quest, the lion representing the first animal to be shot for the Roosevelts^55^.

#### Hunting for Differentiation

The hunting practices of Theodore Roosevelt and Kermit Roosevelt in the colony seem to differ during the colonial mission of 1909. They are the milestones in the emancipation of Kermit Roosevelt^56^. Theodore D. Roosevelt is the first to notice the differentiation of his son in his hunting.

“This left Kermit alone, and he galloped hard on the giraffe's heels, firing again and again with his Winchester. Finally, his horse became completely done out and fell behind; whereupon Kermit jumped off^*^, and, being an excellent long-distance runner, ran after the giraffe on foot for more than a mile. But he did not need to shoot again. The great beast had been mortally wounded, and it suddenly slowed down, halted, and fell over dead. As a matter of curiosity, we kept the Winchester bullets both from Kermit's giraffe and from mine”^57^.

His hunting practices, alone and apart, reveal a progressive distance from his father. They are also permitted by the sustained use of horses in hunting. As such, Kermit Roosevelt exposes an equestrian practice of great experience in spite of his age, so much so that as the mission unfolds, his father gives him his qualities. The equestrian practice of Kermit Roosevelt and his ability also confirm his belonging to the elite culture of the Norths at the beginning of the 20th century (Baratay and Roche, 2015).

The differentiation of Kermit Roosevelt's hunts is also realized thanks to his body and his age. These attributes, although considered as brakes by the hunting community—at least for age—prove to be assets in this new African universe.

“But meanwhile he had run into view of Kermit; and Kermit—who is of an age and build which better fit him for successful breakneck galloping over unknown country dotted with holes and bits of rotten ground—took up the chase with enthusiasm. Yet it was sunset, after a run of six or eight miles, when he finally ran into and killed the tough old bull, which had turned to bay, snorting and tossing its horns”^58^.

As the narrative is written, these adjectives are reinforced in comparison with the adjectives Theodore Roosevelt attributes to himself through writing. Kermit Roosevelt's hunting which is related to the hunt or chase, is contrasted with his father's skill and intelligence. The qualities attributed to the juvenile Kermit are accentuated by this comparative game, as an attempt to present the horizon he still has to travel^59^. Kermit Roosevelt's movement and physical abilities are both praised and criticized. First of all, they are useful for the smooth running of the Roosevelt's mission hunts in Africa. Indeed, they make it possible to run after the hunted animal more than it is capable of^60^. Conversely, they present a criticism of over-movement and inefficient movement, presenting a hunting sports culture of its own. By observing this glance on the young hunter Kermit Roosevelt, we can consider a certain hierarchy as to the good attributes of the big game hunter. In the first place, the address and the intelligence come before any considerations of robustness or bodily strength. These adjectives are discussed at length in Theodore Roosevelt's account of the presentation of the “fine triggers” encountered during their journey. However, for Theodore Roosevelt it is also about presenting his son through his best day in the eyes of his readers. So, there is a double game of reminder of the attributes of the big game hunter on the one hand accompanied, on the other hand, by a laudatory and youthful presentation of his son. This presentation of his son even introduces himself into a more glorious history specific to America.

“Finally, just as he was going into his burrow backward, Kermit raced by and shot him, firing his rifle from the saddle after the manner of the old-time Western buffalo runners.”^61^

This passage is even more interesting because it puts Roosevelt's journey into a reiteration of the conquest of the American West. On horseback, the hunting of buffaloes without support confirms this temporal comparison. The character of Kermit is supposed to recall the history shared around this conquest and the colonization of the Indian populations. It also shows a personification of the juvenile Kermit in this story where he is an allegory (Ricard, 1987).

Moreover, the presence of colonial African hunters, alongside Kermit Roosevelt, tends to help him acquire those skills considered essential in his rite of passage to adulthood and the rank of great hunter. These groups of professional hunters mentor Kermit Roosevelt. They accompany the young Kermit in his empowerment over time areas that become larger and larger as the mission progresses.

Finally, the differentiation of Kermit Roosevelt's hunts is achieved by a numerical progression in the number of animals killed, a true guiding thread of his development^62^. The accounting of the prey killed, their comparative tables, is finally presented in a hunting table at the end of Theodore Roosevelt's book^63^. As conferred by the different hunts of Kermit Roosevelt, he also wants to be the discoverer/hunter of certain new species, supporting his importance in the mission^64^. This differentiation of Kermit Roosevelt is also exposed in his learning of the governments of the colonized. His distance in practice from the patriarchal figure and the skills attributed during the story by his father underline this rise in qualification. Surveillance, government of the colonized and participation in the mission discussions are all signs of this increase in experience of the young Kermit during the mission.

### The Youth of Kermit Confronted With His Progressive Responsibilities in the Practice of Hunting

#### Juvenile Sport Emotions Presented by His Father

The representations presented by Theodore Roosevelt about his son Kermit Roosevelt expose the sporting emotions of a young man facing the perils and torments of hunting in the colony. Under his father's supervision, the young Kermit Roosevelt is given a set of attributes and emotions specific to youth.

“Kermit, who was with Tarlton, galloped the big male, and, although it had a mile's start, ran into it in three miles, and shot it as it lay under a bush. He afterwards shot another, a female, who was lying on a stone kopje. Neither made any attempt to charge. The male had been eating a tommy. The lion was with a lioness, which wheeled to one side as the horsemen galloped after her maned mate. He turned to bay after a run of less than a mile and started to charge from a distance of 200 yards. But Kermit's first bullets mortally wounded him and crippled him so that he could not come at any pace and was easily stopped before covering half the distance. Although nearly a foot longer than the biggest of the lions I had already killed, he was so gaunt—whereas they were very fat—that he weighed but little more, only 400 and 12 pounds.”^65^

Theodore Roosevelt's juvenile sports emotions about his son show an energetic young man whose hunts are a great support. With an overflowing dynamism, the possibilities of exercising, day after day, fill the young Kermit Roosevelt with happiness^66^. The sporting experience is therefore at the center of the juvenile emotions of Theodore Roosevelt's son. The equestrian practices of the young Kermit are representative of his enthusiasm, his strength and his determination. Of the entire missionary cohort, the young man is the one who rides the most miles during the mission. He shows extraordinary resistance, changing, for example, his shooting shoulder when his right shoulder is bruised or recovering from the few pains related to the journey. Whether he is on foot or on horseback, his energy is overwhelming and becomes central. Theodore Roosevelt builds the image of his son around a classical and glorious figure of the 19th century adventurer (Venayre, 2002). Kermit Roosevelt is presented as a holder of all the physical attributes of the adventurer. His physicality is even at the center of his father's discourse. His role tends to categorize him around the figure of the inveterate and insatiable hunter, removing his emotional capacity to step back from his practices and his journey. Moreover, the detour of the Roosevelt mission by Naïrobi allows Kermit Roosevelt to participate in horse races, proof once again of his overflowing energy^67^.

Driven by his dynamism, he sometimes becomes impatient, as for the naturalist discovery which frames the hunting quest. During his hunts and thanks to his enthusiasm, he knows how to respond to exercises outside of hunting by exploring certain caves and African regions. For this point, the link with the African history is frequent:

“Next day, when Kermit and I were out alone with our gun-bearers, we saw another rhino, a bull, with a stubby horn. This rhino, like the others of the neighborhood, was enjoying his noonday rest in the open, miles from cover. “Look at him,” said Kermit, “^*^standing there in the middle of the African plain, deep in prehistoric thought.”^68^

Kermit Roosevelt's view of Africa exposes a retrograde thought that is a complete integration of imperialist theses. In agreement with his father and his representations of Africa, this discourse is illustrated in his descriptions and activities on African soil. Do we need to recall the name of the first chapter of Theodore Roosevelt? Finally, his juvenile emotions and his role in the camp is no less original.

“In the evenings the camp-fires blazed in front of the tents, and after supper we gathered round them, talking or sitting silently, or listening to Kermit strumming on his mandolin.”^69^

Kermit Roosevelt participates in the mission's nightly meetings, which are to be thought of as essential summaries and preparations. His role is however off-center as a musician, although his presence delights the group of hunters around the fire.

The emotions of the young Kermit Roosevelt are therefore those of a young hunter in search of adventure and whose insatiable energy seems to be satisfied with hunting practices. His adventurous and dynamic behavior allows him to gain responsibility in the mission, especially since he is the bearer of a revolutionary practice that tends to hybridize his characterization as a young hunter. This innovation within the scientific mission of the Roosevelt family tends nevertheless to reintegrate him into the hunting fact.

#### A New Photographic Passion: the Young Kermit as His Father's Regular Photographer?

Symbol of technical novelty and distancing from hunting, the young Kermit Roosevelt sees himself as the holder of a technical revolution in the mission supervised by his father, also engaging an imperial gaze toward the whole of Africa he has traveled through (Bancel, 2004). A large part of the photographic records of the mission were in fact entrusted to him, as can be seen in the photographs in Theodore Roosevelt's book and in the collections of the National Museum in Washington^70^. This distancing from the practice of hunting is to be understood in the same movement as the hybridization and use of the term safari by his father. In fact, hunting photography and safari are part of the same shift in the colonial practice of the great African hunts and the animals targeted within them. Are they the result of a collapse of the African fauna forcing a shift in practices? Are they, more simply, the result of a shift in practices toward a more developed range in the face of the easy slaughter permitted by the hunting trip?

“At last Kermit succeeded in getting some good white rhino pictures. He was out with his gun-bearers and Grogan. They had hunted steadily for nearly two days without seeing a rhino; then Kermit made out a big cow with a calf lying under a large tree, on a bare plain of short grass. Accompanied by Grogan, and by a gunbearer carrying his rifle, while he himself carried his^*^” naturalist's graphlex “camera, he got up to within 50 or 60 yards of the dull-witted beasts and spent an hour cautiously maneuvering and taking photos. He got several photos of the cow and calf lying under the tree.”^71^

Because of his younger age and his inferior position of filiation, Kermit Roosevelt is the practitioner chosen to realize the photographic collections. That said, this practice requires more perseverance than hunting practices. It even seems at first to be at the antipodes of the young Kermit's temperament and passion. Moreover, the use of the photographic technique is quite delicate during the hunting practices of the mission since the equipment is rather loud and seems to have a negative impact in the approaches of the hunters. His practice proves to be of central utility for the training of Kermit Roosevelt, in that it allows him to perfect his instruction and to raise his level of placidity and perseverance, dear to his father and to the experienced men of the mission. Sometimes the comparison between his father's double barrel and his camera lasts in the story^72^. It reminds us of the new possibility offered to hunters in their decentering of the hunt. Still, the photographs of the young Kermit give an equal share to the naturalistic photographs compared to those of killed animals. It also recalls the insertion of the photographic technique in a set of scientific, naturalist and hunting research. Didn't he hunt clichés like animals?

Photographing his father's animals and exploits are also an invitation to Theodore Roosevelt's more precise staging in Africa. Indeed, this occupation is not insignificant in that the young Kermit participates in it in a total way. He takes advantage, for example, of a few injuries to develop his photographic practice, in which his father plays an important role. His father appears with all the appearances of the victorious military colonialist. It is a reiteration of the English conquest of East and protectoral Africa^73^. The journey taken by the Roosevelt family, abundantly documented by the pictures of Kermit Roosevelt, is a tangible way of showing this English imperial expansion. Moreover, the use of these roads and forts by the Roosevelts led them, in a way, to participate in this colonial conquest from the southeast of colonial Kenya to the north of protectoral Egypt^74^. The photographic snapshots in which Theodore Roosevelt's son Theodore Roosevelt appears are so many symbiotic relationships created to recall this imperial intermingling between the first American representative in Africa and the English imperial power.

As conferred by the abundance of hunting photographs in which his father sits enthroned in the company of the killed animals, Kermit Roosevelt recovers the task of leaving a visible trace of his father in Africa (Marquis, 2014)^75^. His camera and his photographs are as many possibilities for writing the novel of the United States in colonial Africa. Each photograph participates in this visible writing and opens up the field of possibilities to their media diffusion. Who better than his son to accomplish this task?

#### Emancipating and Governing the Colonized: Kermit's Central Role in the Colonial Government of the Mission

As the Roosevelt's journey and safaris in the colony progressed, Kermit Roosevelt became autonomous from his father and tended to experiment imperial government through increasing exercises on the colonized.

Kermit Roosevelt's mission in colonial Africa allowed him to experience the colonial government of men and spaces through empires. Kermit Roosevelt places himself as administrator of the colonies and as colonist in his empire. He practiced the “government of races.” The young Kermit therefore set about choosing the colonized who should accompany him.

Governing the races and choosing the “savages” who participate in the Roosevelt's mission is of capital importance in the hunting processions on his travels. This training to lead is only revealed in the middle of a missionary campaign. Always accompanied by Leslie Tarlton who finally had to stop his quest for a few days, Kermit had to choose a new colonized auxiliary in the person of Juma Yohari^76^.

“Kermit's two gun-bearers were Juma Yohari, a coal-black Swahili Moslem, and Kassitura, a Christian negro from Uganda. Both of them were as eager to do everything for Kermit as mine were to render me any service, great or small, and in addition they were capital men for their special work. Juma was always smiling and happy and was a high favorite among his fellows^77^.”

The empowerment of Kermit Roosevelt passed through the engagement of several colonized people whom he placed under his hunting command. However, these colonized people are intermediate figures of colonization in the sense that they are part of the top of the pyramid of colonized people (Glasman, 2010). Although the Roosevelts label them racializing qualifications, they are not only nameless carriers to whom R. J. Cuninghame would like to give numbers to better know them. They are named by a surname and sometimes a first name. In addition to their salaries, they receive symbolic gifts that show their importance in the Roosevelt mission.

“Kassitura, quite as efficient and hard-working, was a huge, solemn black man, as faithful and uncomplaining a soul as I ever met. Kermit had picked him out from among the porters to carry his camera and had then promoted him to be gun-bearer. In his place he had taken as camera-bearer an equally powerful porter, a heathen “Mnuwezi named Mali”. His tent-boy had gone crooked, and one evening, some months later, after a long and trying march, he found Mali, whose performance of his new duties he had been closely watching, the only man up; and Mali, always willing, turned in of his own accord to help get Kermit's tent in shape, so Kermit suddenly told him he would promote him to be tent-boy.”^78^

These intermediate figures seem to be of central importance in learning about Kermit Roosevelt's government. The “Bwana Medogo”—Young Master—participates in post advancements for the colonized under his tutelage, going here from bearer of the Graphlex to auxiliary to his person^79^. These recruitments on the various African territories crossed by Kermit Roosevelt reveals his emancipation by the colonial government. The young Kermit is surrounded by a colonial rearguard at his service. These recruitments of privileged intermediaries linked to Kermit Roosevelt expose more precisely a pyramid of advancements from the tent boy to the Saïs, passing through the various inalienable individualized carrier posts of the long hunting caravans on their travels^80^. The Saïs remain the cornerstone of the Roosevelt's hunting practices in that they guide the white hunters to their prey (Ramaswamy, 2009).

## Conclusion

This paper is intended to be a first contribution to the history of elite filiations in sport, a lineage between Theodore Roosevelt and his son Kermit Roosevelt in English East Africa. The framework of the scientific mission for which the former president of the United States is in charge, engages the young Kermit in a striking training in African hunting and an initiation to colonial government. The education of Theodore Roosevelt's son takes place in a theoretical teaching of literary studies, in the framework of his own personal development, and in the military matter framed by networked actors who support him throughout his quest. The scientific commission entrusted to the Roosevelts remains a central influence in the course of their African journey and safaris. However, it allows their filiation to be built up throughout the mission while allowing the young Kermit Roosevelt to gradually emancipate himself. Central for Kermit Roosevelt, sport hunting allows him to develop different skills around a photographic and governmental diptych to match his growing experience.

In a more general way, this paper could propose the opening of more important research on the fields of filiation and emancipation in sport. It could include longitudinal studies on different social classes of origin and different spaces of practice, and the moments of induction into sport by filiation could be compared.

The article proposes an analysis of the elite knowledge and techniques transmitted by lineage on the English African colonial territory and not of the effects of the voyage organized by Theodore Roosevelt for his son Kermit Roosevelt. This evaluation of training for exploration and international peregrinations could, as such, give rise to new research. In this logic, the access to archives of the intimate would certainly bring new questionings as for this research. These archives could also make it possible to broaden the subject of analysis to the entire Roosevelt family.

This article opens the way to a global comparative study on the filial relations between cosmopolitan elites. More precisely, it is the analysis of the modalities of learning knowledge and techniques in different colonial or postcolonial spaces that arouses the most interest. More precisely, our interest would turn to the analysis of the role of sport - including hunting—in the social and intellectual construction of cosmopolitan elite lineages. The permanence of the socio-political status of the elites could thus be assessed through the prism of these research criteria and their groups of belonging or their international movements. We could also—which an article of this size does not allow—dwell on the effects of this longitudinal training in the medium and long term in the lives of the heirs of these elites. Thus, it will be necessary to establish a comparable and comparative grid according to criteria that can be accepted by all the territories from which these groups or individuals are extracted. We could draw inspiration from the prosopographical analysis of the group of researchers led by Patrick Clastres in order to set up a global comparative study between different areas of origin of these elites, where hunting could be the anchor point of this future international research^81^. Finally, and as a mirror to this introductory statement, this article proposes an opening onto the strategies of social classes struggles, such as the bourgeoisie with international elites that could mimic this formation, and eventually integrate and penetrate the positions of these same elites.

## Data Availability Statement

The original contributions presented in the study are included in the article/supplementary material, further inquiries can be directed to the corresponding author.

## Author Contributions

The author confirms being the sole contributor of this work and has approved it for publication.

## Conflict of Interest

The author declares that the research was conducted in the absence of any commercial or financial relationships that could be construed as a potential conflict of interest.

## Publisher's Note

All claims expressed in this article are solely those of the authors and do not necessarily represent those of their affiliated organizations, or those of the publisher, the editors and the reviewers. Any product that may be evaluated in this article, or claim that may be made by its manufacturer, is not guaranteed or endorsed by the publisher.

## Footnotes

^1^See for this point John Mackenzie's seminal work on this subject in his book: Mackenzie J. *The Empire of Nature: Hunting, Conservation and British Imperialism*, Manchester: Manchester University Press (1988). 350 p.; and the work of Steinhard E. I., *Black Poachers, White Hunters. A Social History of Hunting in Colonial Kenya*, Oxford: Ohio University Press (2006). 248 p.; and of Thompsell A. *British Sport, African Knowledge and the Nature of Empire*, London: Palgrave Macmillan (2015). 229 p.

^2^See the ages of Th. Roosevelt Jr., P. of Savorgnan de Brazza or of E. Bruneau de Laborie during their government missions.

^3^Theodore Roosevelt in his 1913 mission to Brazil also left with Kermit Roosevelt: Roosevelt T. *Through the Brazilian Wilderness*, New York: Charles Scribner's Sons (1914). 383 p.

^4^See Roosevelt T., *op cit*.; Smithsonian Institution, “Digital archives”, Collections Search Center, https://collections.si.edu/search/ [accessed March 1, 2019]; American Museum of Natural History, “Digital archives”, Digital Special Collections, http://lbry-web-007.amnh.org/digital/index.php [accessed March 1, 2019]; Theodore Roosevelt Center*, “*Digital archives”, Digital Library, Dickinson State University, Dickinson, https://www.theodorerooseveltcenter.org/Research/Digital-Library [accessed March 1, 2019] and the printed sources of the French-language newspapers and magazines: GALLICA, BNF, “Archives digitales”, https://gallica.bnf.fr/services/engine/search/advancedSearch/?lang=fr [consulted on March 1, 2019].

^5^Roosevelt T. *African Game Trails*. New York: Charles Scribner's Sons (1910). p. 523.

^6^*Op.cit*. p. 24.

^7^See, for example, Bruneau de Laborie E. *Guide de la chasse et du tourisme en Afrique centrale*. Paris: Société d'éditions géographiques, maritimes et coloniale*s* (1931). p. 9-18.

^8^Roosevelt T. *African Game Trails*. New York: Charles Scribner's Sons (1910). p. 527.

^9^See Stanton T., “Lettres américaines,” Mercure de France (February 1, 1911). p.654-659 and the excerpts “Sur la piste du gibier africain” from *African Game Trail*, published in *Le Journal* (September 28, 1909).

^10^Roosevelt T. *African Game Trails*. New York: Charles Scribner's Sons (1910). p. 522.

^11^*Op.cit*. p. 24.

^12^*Op.cit*. p. 160.

^13^*Op.cit*. p. 362.

^14^*Op.cit*. 522-523.

^15^*Op.cit*. p. 523.

^16^*Op.cit*. p. 24.

^17^*Op.cit*. p. 522.

^18^*Ibidem*.

^19^*Op.cit*. p. 57-79.

^20^*Op.cit*. p. 24.

^21^See also: Chasles R. (2015) *Politique de trois chasseurs en voyage, Philippe d'Orléans, Henri d'Orléans et Ferdinand D'Orléans - 1879-1912* [Master's thesis] 200 p.; and see, in a non-exhaustive manner, the works of Foa E., Selous F. C., Rawdon M. and Bruneau de Laborie E. on the question of the presentation of weapons on hunting trips.

^22^Roosevelt T. *African Game Trails*. New York: Charles Scribner's Sons (1910). p. 22-23.

^23^*Op.cit*. p. 118.

^24^*Op.cit*. p. 23.

^25^*Op.cit*. p. 396.

^26^*Op.cit*. p. 24.

^27^*Op.cit*. p. 3.

^28^*Op.cit*. p. 483.

^29^Pease A. “A Volcanic Crater in Northern Somaliland”. *The Geographical Journal* (1898) 11:2:138-142; and Pease A. *The Book of the Lion*, London: St. Martin's Press (1987). 291 p.

^30^Roosevelt T. *African Game Trails*. New York: Charles Scribner's Sons (1910). p. 72.

^31^*Op.cit*. p. 77.

^32^*Op.cit*. p. 221.

^33^Richmond C.W. “In Memoriam: Edgar Alexander Mearns”. *The Auk* (1918) 35:1-18.

^34^Dr. Mearns is an experienced man and has been involved in numerous military campaigns. He participated in the U.S. military campaign in the Philippines from 1899 to 1902.

^35^Roosevelt T. *African Game Trails*. New York: Charles Scribner's Sons (1910). p. 137.

^36^Heller E. “Obituary Notices”. *Journal of Mammalogy* (1939) 4:529.

^37^Roosevelt T. *African Game Trails*. New York: Charles Scribner's Sons (1910). p. 5-6.

^38^*Op.cit*. p. 18.

^39^Four of these professional hunters organizing the mission participated in the Boer War. The Boer War led to their departure and their reorientation towards the naturalist quest and the professional hunting activity, both of which are difficult to dissociate.

^40^Roosevelt T. *African Game Trails*. New York: Charles Scribner's Sons (1910). p. 3.

^41^Author-free. “TR's arrival in Africa.” Washington D.C.: Library of Congress (1909) 00:20; Shaw A. *A Cartoon History of Roosevelts Career*. New York: The Review of Reviews Company (1910). 254 p.

^42^Roosevelt T. *African Game Trails*. New York: Charles Scribner's Sons (1910). p. 137.

^43^*Op*.*cit*. p. 29.

^44^*Op.cit*. p. 17.

^45^*Op.cit*. p. 53.

^46^*Op.cit*. p. 415.

^47^*Op.cit*. 137.

^48^*Op.cit*. p. 483.

^49^*Op.cit*. p. 20.

^50^*Op.cit*. p. 28.

^51^*Op.cit*. p. 129.

^52^*Op.cit*. p. 61-62.

^53^See, for example, the famous stories of lion hunting: *Op.cit*. p. 47-49.

^54^*Op.cit*. p. 75.

^55^*Op.cit*. p. 57- 79.

^56^*Op.cit*. p. 28.

^57^*Op.cit*. p. 98.

^58^*Op.cit*. p. 28.

^59^*Op.cit*. p. 177.

^60^*Op.cit*. p. 76.

^61^*Op.cit*. p. 202.

^62^*Op.cit*. p. 319.

^63^*Op.cit*. p. 457-458.

^64^*Op.cit*. p. 215.

^65^*Op*.*cit*. p. 159-161.

^66^*Op*.*cit* p. 191.

^67^*Op*.*cit*. p. 225-226.

^68^*Op*.*cit*., p. 172.

^69^*Op*.*cit*. p. 169.

^70^Theodore Roosevelt takes a few naturalist pictures as well.

^71^*Op*.*cit*. p. 412-413.

^72^*Op*.*cit*. p. 192.

^73^*Op*.*cit*. p. XVIII.

^74^*Op*.*cit*. p. 279.

^75^*Op*.*cit*. p. I; p. 208; p. 262.

^76^*Op*.*cit*. p. 315.

^77^*Op*.*cit*. p. 332.

^78^*Ibidem*.

^79^*Op*.*cit*. p. 99.

^80^*Op*.*cit*. p. 332.

^81^CLASTRES P., CARPENTIER F., KLIMA H., *Les membres du Comité international olympique de 1894 à 1972, Prosopographie d'une élite transnationale*, FNS Project, 2018-2022.
